# Organic Functionalized Carbon Nanostructures for Solar Energy Conversion

**DOI:** 10.3390/molecules26175286

**Published:** 2021-08-31

**Authors:** Luca Lazzarin, Mariacecilia Pasini, Enzo Menna

**Affiliations:** 1 Department of Chemical Sciences & INSTM, University of Padua, Via Marzolo 1, 35131 Padova, Italy; luca.lazzarin@phd.unipd.it; 2 Institute of Chemical Sciences and Technologies “G. Natta”-SCITEC, National Research Council, CNR-SCITEC, Via Corti 12, 20133 Milan, Italy; 3 Interdepartmental Centre Giorgio Levi Cases for Energy Economics and Technology, University of Padua, 35131 Padova, Italy

**Keywords:** carbon nanotubes, graphene, reduced graphene oxide, graphene quantum dots, organic functionalization, dye, organic photovoltaics, dye-sensitized solar cells, perovskite solar cells, photocatalytic hydrogen evolution

## Abstract

This review presents an overview of the use of organic functionalized carbon nanostructures (CNSs) in solar energy conversion schemes. Our attention was focused in particular on the contribution of organic chemistry to the development of new hybrid materials that find application in dye-sensitized solar cells (DSSCs), organic photovoltaics (OPVs), and perovskite solar cells (PSCs), as well as in photocatalytic fuel production, focusing in particular on the most recent literature. The request for new materials able to accompany the green energy transition that are abundant, low-cost, low-toxicity, and made from renewable sources has further increased the interest in CNSs that meet all these requirements. The inclusion of an organic molecule, thanks to both covalent and non-covalent interactions, in a CNS leads to the development of a completely new hybrid material able of combining and improving the properties of both starting materials. In addition to the numerical data, which unequivocally state the positive effect of the new hybrid material, we hope that these examples can inspire further research in the field of photoactive materials from an organic point of view.

## 1. Introduction

Research on organic photovoltaics (OPVs) exploded at the beginning of the millennium thanks to the use of fullerene derivatives as electron acceptors, coupled with conjugated polymers as donor materials [1,2,3]. Development of OPVs actually followed the discovery of fullerenes, and in particular, took off as soon as the first efficient functionalization approaches became available.

Since then, the evolution of the field of non-conventional or third-generation photovoltaics have introduced new families of devices, such as dye-sensitized solar cells (DSSCs), perovskite solar cells (PSCs), and photocatalytic cells, based on different active materials, but sometimes also reinventing the role of fullerenes [4]. At the same time, new carbon allotropes, collectively named carbon nanostructures (CNSs), have been explored as components with the aim to improve energy-conversion efficiency or stability of devices [5,6,7,8,9,10,11,12,13].

Indeed, the superior properties of CNSs, such as metallic or semiconducting electronic behavior, thermal conductivity, and surface area, can contribute to the enhancement of solar conversion into electricity or into production of fuels through photocatalysis. Although many examples based on pristine CNSs have been reported, the present review is focused on the contribution of organic chemistry in this field, as reported in the most recent literature. In fact, organic moieties can modify CNSs to provide or modify fundamental properties for the development of energy-conversion applications, such as light absorption, electron transfer, charge transport, solubility, and interaction with a polymer matrix [14].

## 2. Chemistry of Carbon Nanostructures

The family of CNSs includes carbon nanotubes (CNTs), either single- or multi-walled (SWCNTs and MWCNTs, respectively), graphene-based materials (GBMs), carbon dots, and graphene quantum dots (GQDs). The most common GBMs, in turn, are graphene, graphene oxide (GO), and reduced graphene oxide (RGO), although there is no sharp distinction among them. In fact, a continuous variation of structural features is found in GBMs, involving the density of oxygenated groups (O/C atomic ratio), lateral size of sheets, and number of layers [15].

Covalent and non-covalent functionalization strategies have different advantages and drawbacks. While covalent chemistry is based on a stable linkage, providing good solubility and ensuring a closer proximity of the active moiety to the CNS, thus enabling efficient electron-transfer processes, supramolecular or non-covalent approaches avoid the need of complex reactions and purifications involving CNS suspensions. In fact, once the proper molecular structure is obtained through conventional synthesis, a simple mixing with the suspended CNS is required. Moreover, covalent chemistry involves the introduction of sp^3^ defects in the sp^2^ lattice of CNTs and graphene that can often have a negative impact on the electrical properties of the material, while non-covalent strategies have been developed that allow the improvement of the CNSs’ processability without degrading electronic features [16].

Some covalent strategies are based on carboxylic groups or other oxidized moieties, while others rely on specific reactions taking place on the sp^2^ carbon lattice.

Among the latter family, the 1,3 dipolar cycloaddition of azomethine ylides, one of the most widely used reaction of fullerenes [17], was adapted for the functionalization of CNTs [18] and graphene [19]. This is known as the Prato reaction. The reactive azomethine ylide is usually obtained in situ through decarboxylation of an immonium salt, derived from the condensation of an α-amino acid with an aldehyde. Cycloaddition of the ylide to a double bond of the nanostructure affords a pyrrolidine ring fused to the CNS. The commercial availability of a wide range of carbonyl and amino acid precursors and the possibility to obtain more through simple chemical transformations makes this reaction an extremely versatile and still widespread functionalization tool [20,21]. In fact, a proper choice of precursors allows the introduction of one or more desired residues on the derivative, as substituents on the different positions of the pyrrolidine ring.

Diazonium chemistry provides an efficient means for the functionalization of CNTs and GBMs, through the so-called Tour reaction, after the name of the researcher who first introduced it [22]. The diazonium salt, either previously isolated or generated in situ, starting from an aniline precursor, undergoes reductive dissociation with loss of N_2_ and formation of an aryl radical that reacts with C=C double bonds of the CNS. The reaction is quite fast and can afford derivatives densely decorated with aryl moieties or even with branched oligomeric structures when pushing reaction conditions [23]. In general, starting with an aniline with the desired moiety in position 4, it is possible to obtain a CNS decorated with the corresponding phenyl derivative in a fast and effective way [24,25].

As first proposed by Smalley and coworkers in 1998 [26], carboxylic groups can be formed on CNTs through oxidation and then used to form amide or ester bonds with molecules bearing amine or alcohol groups, respectively. Different oxidation approaches have been proposed, although the most commonly used is based on treatments with strong acid mixtures, such as concentrated HNO_3_/H_2_SO_4_. As with usual organic synthesis, amidation can follow different activation strategies, such as the formation of acyl halide intermediates or the use of carbodiimide coupling agents. The same approach can be adapted to all CNSs, taking in account that in the case of GO, carboxylic groups are already present in the structure, and also has found application in the recent literature [27,28].

Non-covalent functionalization takes advantage of the sp^2^ carbon surface of CNSs to establish hydrophobic interactions in general, consisting mainly of π–π interactions with large aromatic structures [29] including a number of pyrene derivatives [30,31,32], but also conjugated polymers [33,34] and porphyrins [35]. Other non-covalent interactions have indeed been found, [16,36,37,38] including XH–π, anion/cation–π, and CH–π, extending the range of structures that can interact with CNSs to molecules and polymers bearing charges and/or many different functional groups.

A peculiar non-covalent approach, in the case of CNTs, consists of the encapsulation of molecules inside the tubes to afford the so-called peapods, first introduced by Luzzi with fullerenes [39]. A typical encapsulation procedure is based on the diffusion of molecules in the vapor phase at high temperature and low pressure, or from solution or even by means of supercritical carbon dioxide [40]. The approach also was more recently extended to a range of photoactive organic molecules [41].

Besides common experimental techniques for the detection of covalent bonds, such as FT-IR and NMR (that in the case of CNS derivatives hardly find application), typical characterizations of materials can effectively provide evidence of CNS functionalization. Thermogravimetric analysis (TGA) in particular allows researchers to quantify the functionalization degree (FD), defined as the fraction of CNS carbon atoms that are functionalized with respect to the total carbon atoms, by comparing the weight loss due to the organic fraction with that of the nanostructure (occurring at higher temperatures) [42].

On the other hand, experimental evidence of non-covalent interactions for aromatic molecules and quantification of association constants can be provided by fluorescence measurements [43,44].

## 3. Dye-Sensitized Solar Cells

Since the first discovery of a dye-sensitized solar cell (DSSC) by Grätzel and O’Regan [45], this technology has drawn the interest of scientists from all over the world. Thanks to a new cost-effective approach to the energy conversion, this scalable, versatile device finds its application in a different area with respect to the usual silicon-based cell. While thorough and exhaustive descriptions of the generic aspects of the DSSC device and its specific architecture can be found in the literature [45,46,47], we will briefly recall the basic concepts here. In general, a molecular sensitizer (dye) adsorbed on an inorganic semiconductor (TiO_2_) is excited by solar light, giving rise to a subsequent electron injection from the dye to titania. The oxidized sensitizer is regenerated by a redox mediator (usually a I_3_^−^/I^−^ redox couple) dissolved in an electrolyte.

Percolation through TiO_2_ and then to the anode feeds electron circulation in the external circuit, ending with the injection from the cathode to redox mediator in the electrolyte, thus closing the circuit.

In an effort to minimize the cost and the environmental impact, recent advances have been reported [48] regarding the substitution of the ruthenium-based dyes with metal-free organic molecules and the principle in the design of this new class of sensitizers [49]. This review begins with the metal-free approach, but proposes a further insight taking into account hybrid materials that spring from the co-use of CNSs and organic moieties as a photoactive element.

There are mainly three unwanted processes: back electron transfer (1), in which the photoinjected electron interacts with the oxidized state of the sensitizer or the oxidized state of redox couple; excited-state deactivation, consisting of radiative (2) and/or non-radiative (3) paths involving the dye.

In order to minimize the detrimental effect on the overall performance, it is necessary to elaborate suitable improvements regarding the architecture of the cell itself and the materials used. Several strategies have been developed to optimize the overall efficiency, including the use of carbon-based materials, which so far have shown promising effects.

The very first successful inclusion of a CNS in a DSSC has paved the way to a new concept in the design of the solar cell. At first, the synergistic use of graphene-materials regarded mainly the construction of transparent electrodes, semiconducting layers and counter-electrodes, as already exhaustively reported [5,50], and the specific use of carbon nanotubes in the construction of DSSCs has been studied and reviewed as well [51]. However, the only use of a carbon nanostructure reported before 2010 was as a pristine sensitizer of a semiconductor, in the form of quantum dots [52]. The idea behind this application lies in the high electron mobility and in the strong and broad absorption profile of pristine graphene, which is able to absorb around 2.3% of light for each single carbon layer [53]. Unfortunately, the performance generated by cells with pristine graphene was quite low (η = 0.06%), but since then, several improvements have been introduced, giving rise to a new class of hybrid materials.

For instance, in a recent publication, Gatti et al. proposed a new hybrid dyad DSSC photosensitizer using RGO and a donor–π–acceptor system [54]. The organic unit attached was triphenylamine (D)-thiophene (π)-cyan acrylic acid (A), covalently bound thanks to the Tour reaction. The molecule shows a relevant absorption of visible light due to the extended π-conjugation, which allows an efficient push–pull effect of photoexcited charges throughout the molecular backbone.

**Figure 1:**
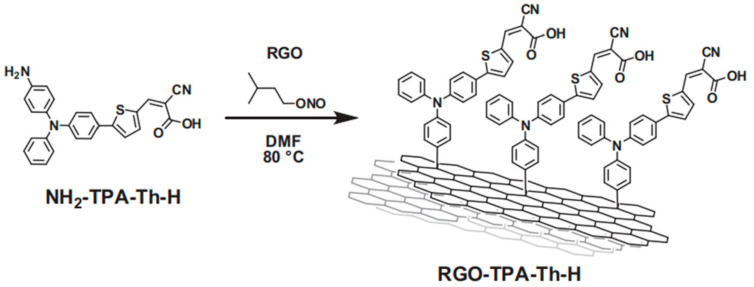
Functionalization of RGO with the donor–π–acceptor system to furnish the new hybrid species. Reproduced from [54] with the permission of Elsevier.

A schematic representation is reported in Figure 1.

Covalent binding was demonstrated by a bathochromic shift in comparison with the free dye (TPA-Th-H): the π–π* transition shifted from 303 to 308 nm, and the internal charge transfer ICT peak from 409 to 411 nm, similar to other chromophores reported in the literature [55]. A further proof can be found in the Raman spectrum. The different I_D_/I_G_ ratio in comparison with pristine RGO demonstrates that the RGO defects are modified by means of the covalently introduced organic moieties. The performance of the cell was not high, which can be attributed to a low loading of the TPA-Th-H unit, but harsh desorption experiments (1 M NaOH in DMF at 70 °C for 7 days) highlighted the chemical stability of the new hybrid system.

**Figure 2:**
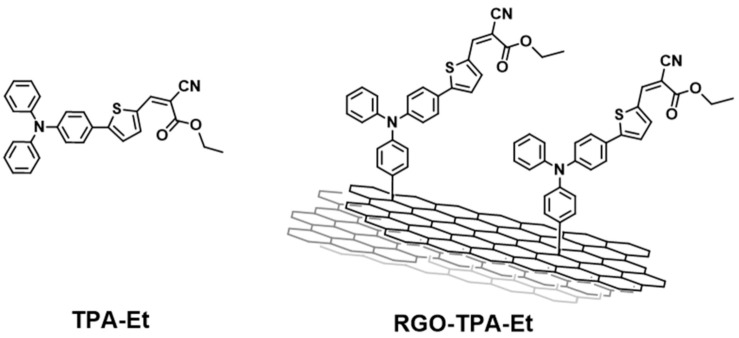
Structure of the reference-free dye TPA-Et (**left**) and hybrid material RGO-TPA-Et (**right**) reported in ref. [56].

The photophysics of a similar system was reported [56], allowing a few interesting considerations about the role of the RGO in a photoactive push–pull system. The hybrid under examination is shown in Figure 2.

Even in this case, the bathochromic shift of 3 nm between the free dye (406 nm) and the dyad (409 nm) testified to the covalent bond.

Steady-state photoluminescence (in particular in the region of 500–700 nm) measurements were performed, showing an evident quenching of the emission of methyl-2-cyano-3-(4-(diphenylamino)phenyl)acrylate (TPA-Et) when attached to RGO. Time-resolved photoluminescence experiments confirmed the faster photoluminescence decay of the hybrid in comparison with the free TPA-Et. These data therefore suggested that the photoinduced charge separation is rapidly quenched in presence of RGO, and highlighted the high mobility of electrons between the π-system and the inorganic substrate. Additionally, electron paramagnetic resonance (EPR) measurements of the hybrid system and the reference both attached to TiO_2_ demonstrated unequivocally that the electron transfer from TPA-Et to the semiconductor takes place, even in the presence of RGO. It is therefore legitimate to assume that the RGO acts like an electron reservoir, being able to efficiently reduce the oxidized state of TPA-Et and consequently helping the regeneration of the sensitizer. Moreover, given the tendency of the TPA-Et radical cation to dimerize, the presence of a substrate capable of the immobilization of the dye should be beneficial for the long-term stability of the cell under operating conditions. In addition, the steric hindrance of a bulky substrate such as the RGO grants a lesser π-stacking, which is a common problem that affects photovoltaic performance [57].

**Figure 3:**
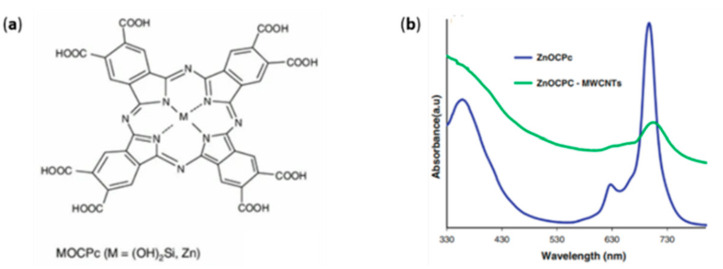
Structure of the MPc (**a**), and absorbance behavior of the pristine ZnPc and its hybrid (**b**). Reproduced from [58] with the permission of the American Chemical Society.

A different approach to the functionalization of MWCNTs concerns the formation of a dyad thanks to the introduction of reactive moieties on the surface of the CNS. For instance, a metallo-octacarboxyphthalocyanine (MOCPc) has been reported as a hybrid-sensitizer in a DSSC [58]. Metallophthalocyanines (MPc) are complexes containing non-transition metals, such as Zn or Si(OH)_2_, placed in a wide π-system. These 18 π-electron aromatic porphyrins are limited as a photoactive element due to MPc dye aggregation, electron recombination with oxidized dye molecules, and a lack of electronic connectivity with the surface of the semiconductor. In order to overcome these limitations, a covalent hybrid with MWCNTs has been synthesized, the spectroscopic absorption of which is reported in Figure 3.

The spectrum collected in DMF showed two absorptions in the visible range at 680–700 nm due to the monomeric species and at 620 nm due to the dimeric species. The introduction of the CNTs caused a red shift of the main peak, in accordance with the attachment of an electron-withdrawing group such as the amine-functionalized CNT.

**Figure 4:**
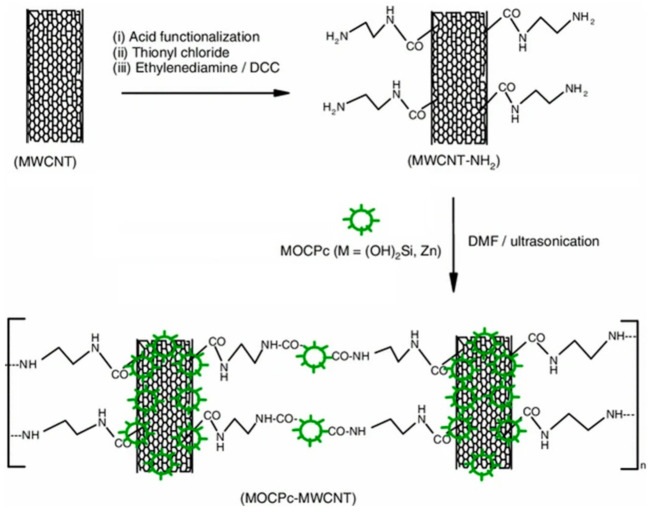
Synthetic route for the MOCPc–MWCNT hybrid. Reproduced from [58] with the permission of the American Chemical Society.

An interesting approach was adopted in this work, especially in comparison with the others previously reported, because the MWCNT was pretreated and then, after the attachment of reactive groups, was covalently bound to the MPc, as shown in (Figure 4).

The so-synthesized hybrid was further studied from a photo-electrochemical point of view in a typical DSSC. The effect of the introduction of the CNS can be discussed in terms of photo-electrochemical impedance spectroscopic studies and photo-chronoamperometric studies. The latter analysis showed a rectangular shape for the free sensitizer in response to the on/off light, which can be attributed to an intimate contact between the electrolyte and the MOCPc. The same conclusion can be made for the hybrid, but, in addition, we wanted to highlight the enhanced photocurrent after the introduction of the MWCNTs, equal to an improvement of 36%. The positive effect on the photocurrent could be a consequence of a better light scattering by the MWCNTs in proximity of the TiO_2_/electrolyte interface [59]. Electro-impedance measurements are an effective tool for the investigation of the electro-transport and recombination mechanisms that take place in a DSSC. The Nyquist plots showed a semicircle that was three times smaller for the MWCNT–MPc, which was clearly related to a significant reduction in the charge-transfer resistance at the TiO_2_/MPc-dye/electrolyte interface. Thanks to the relatively high-conductivity characteristic of the MWCNTs, the photopromoted electrons could rapidly diffuse into the embedded CNS, resulting in a lower overall resistance and a higher photocurrent density. Once again, the synergy between organic chemistry and chemistry of materials led to a better hybrid that overwhelms the individual elements of itself.

**Figure 5:**
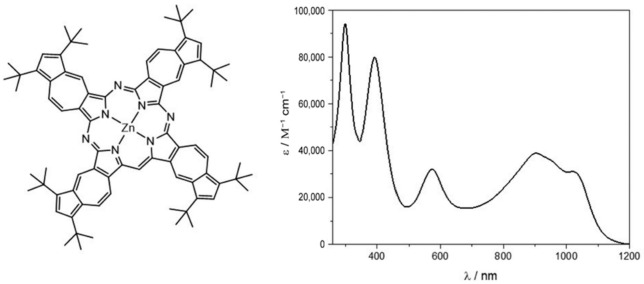
Azulenocyanine π-extended system and absorption spectrum. Reproduced from [60] with the permission of the Royal Society of Chemistry.

Another similar complex consisting of an NIR-absorbing azulenocyanine and a few layers of graphene has been reported [60]. In this case, the wide aromatic core of the azulenocyanine promoted the exfoliation of graphene thanks to effective π–π interactions with the basal plane of graphene. A schematic representation is shown in Figure 5.

The UV–vis–NIR spectrum shows the characteristic absorbance of the molecule under examination. Focusing on the visible range, two main peaks dominated the spectrum around 400 and 580 nm. The former resembled the Soret band absorption of phthalocyanine, the latter the Q-band. Additionally, a wide absorption was present in the NIR region, ranging from 800 to 1100 nm. These spectroscopic features made the complex under analysis a suitable candidate for a DSSC application. Electrochemical measurements pointed out the better behavior as electron donor of the azulenocyanine, while pump probe experiments highlighted the electron transfer from the π-system to graphene upon photoexcitation. The concomitance of these two features makes the sensitizer a promising candidate for its use in DSSCs. Both the free dye and the hybrid system were used as sensitizers in a DSSC, but unfortunately, it turned out that voltage and current density were low, which could be ascribed mainly to two factors: firstly, the lack of carboxylic acid, which allowed a strong binding to TiO_2_; and secondly, the driving force promoting the photoinjection of the electron was very low due to energetic disposition of the lowest unoccupied molecular orbital (LUMO) in comparison to the lower edge of the conduction band. Nonetheless, it was proven that the hybrid system generated better performance than the free azulenocyanine.

**Figure 6:**
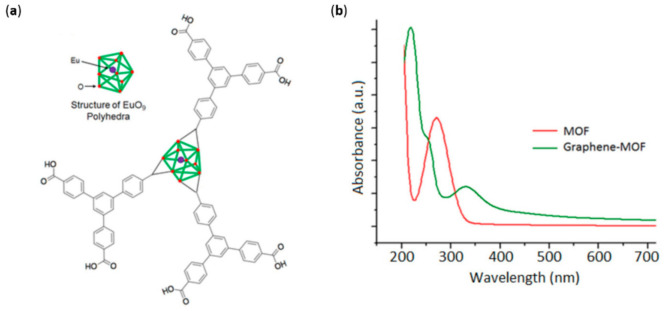
Structure of Eu–MOF (**a**). Absorbance profile (**b**) of the pristine MOF and the hybrid MOF–graphene. Reproduced from [61] with the permission of Elsevier.

Another peculiar system was reported by Kaur et al. [61] involving the immobilization of a metal–organic framework (MOF) on a graphene sheet. To begin with, MOFs are structures hierarchically organized that mimic the structure of natural entities such as chloroplasts [62]. They can be synthesized from different organic linkers and metal centers, but a common feature is a high surface area, which efficiently absorbs incident light and makes them suitable for an energy-conversion system. The idea behind their use as a sensitizer in DSSCs lies in the ability of these structures to absorb light and photoinject electrons in TiO_2_ films, as proven in other works [63]. The structure and absorption spectrum of the MOF under examination, based on a EuO_9_ polyhedron, is reported in Figure 6.

The spectrum underlined the strong UV absorbance around 260 nm of the MOF due to the contribution of the ligands. When combined with graphene instead, a new peak around 250 nm appeared, which could be attributed to η-π* transition of the C-O bond [64]. The absorbance spectrum, concurrently with FT-IR and Raman measurements, demonstrated the successful inclusion of the MOF in a graphene sheet thanks to a simple electrochemical deposition. In fact, the structure remained intact after the electrodeposition, and the interaction binding the Eu–MOF to graphene could be identified in the π–π stacking. The concurrent use of graphene can make up for the characteristic insulating nature of the MOF–Eu (conductance of the order of 10^−12^ S cm^−1^); in fact, the novel material showed an efficient electron collection and an improved conductance (300 mS cm^−1^). Specifically, even if MOF can act as a photoactive material, it cannot conduct electricity. However, a MOF–graphene composite has proven to efficiently inject electrons in the conduction band of TiO_2_. In conclusion, a thin-film graphene–MOF was proven in a lab-scale DSSC device, reaching a power conversion efficiency (PCE) of 2.2%, which was by far better than that of the previously reported MOF-based DSSC [65].

Reported uses of hybrid MOFs involve not only the photoanode, but also the modification of the counter electrode. For instance, to cite the most recent literature, a counter electrode based on transition metal selenides in a MOF interconnected by CNTs has been published [66]. In addition, another example concerns the use of copper polypyrrole-functionalized MWCNT nanocomposites obtained through the electrodeposition technique [67].

**Figure 7:**
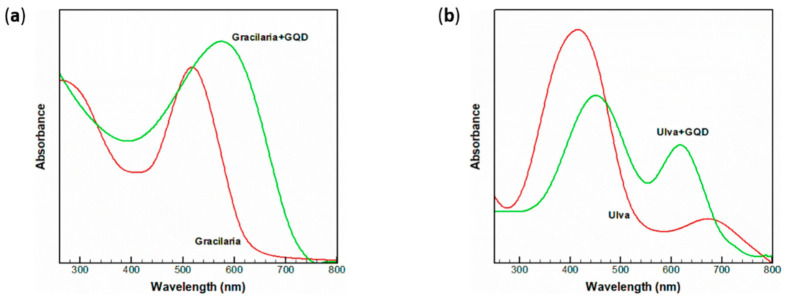
Absorbance spectrum in ethanol of Gracilaria and its hybrid material (**a**), as well as Ulva (**b**). Reproduced from [72] with the permission of Springer.

Moving towards an eco-friendly approach, the use of natural dyes obtainable directly from natural sources has spread over the past years [68]. This trend perfectly suits the desired low-cost and environmental low-impact features of DSSC technology. In particular, a specific attention has been focused on totally renewable sensitizers derived from seaweeds and algae [69,70,71]. In this regard, we highlight a hybrid system stemming from the co-use of GQD and green and red algae [72]. Both algae were treated with a simple extraction method, and the resulting dye was a mixture of the photoactive components present in the seaweed. The red alga, named Gracilaria, showed an absorption characteristic of Phycobilins, while the green one, known as Ulva, was characterized by spectroscopic features of the chlorophyll family. Both were tested as photoactive elements, as a pristine molecule and as a hybrid material, in a conventional DSSC set-up. We report the absorption spectrum of the sensitizers and their respective hybrids in Figure 7.

Two main absorptions in the near-visible region are presented in Figure 7 for the dyes extracted from Gracilaria and Ulva, respectively, at 530 and 420 nm. The visible light absorption could be attributed to the excitation from the σ and π orbital to the lowest unoccupied molecular orbital. Since Gracilaria is characterized by a broader absorption in the visible region, it is expected to be a better chromophore in its energy-conversion application. Subsequently, the introduction of graphene quantum dots could be estimated in reference to the absorption spectrum. In both cases, there was a widening and a red-shift of the peak that has been reported in the literature as a direct effect between the nanostructure and the dye [73]. The introduction of GQDs is thought to alter the disposition of the HOMO-LUMO orbitals, consequently resulting in a faster electron extraction, which has a beneficial impact on the overall performance of the cell [74]. This statement was proved in this work as well: the efficiency of the DSSC using pure Gracilaria rose from 0.52% to 0.94% after the introduction of GQDs. In a similar manner, in the case of Ulva, it was improved from 0.39% to 0.81% following the co-use of the CNS. Photoluminescence measurements showed a severe quenching after the addition of GQDs, and as previously reported and discussed, this could be a proof of a close contact in terms of electron exchange. The radiative path from the excited state to the ground level was inhibited by the presence of the CNS, meaning a reduction in electron-hole recombination from the photoconversion point of view.

**Figure 8:**
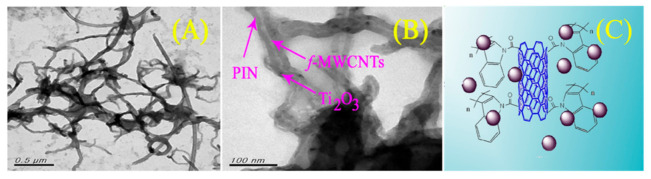
TEM images of an f-MWCNTs-PIN/Ti_2_O_3_ nanocomposite (**A**), its detail (**B**), and illustration of the nanocomposite hybrid f-MWCNTs-PIN/Ti_2_O_3_/TiO_2_ (**C**). Reproduced from [75] with the permission of Springer.

Another useful application of a CNS, reported by Sireesha et al. [75], regarded the synthesis of a hybrid nanocomposite and its use as photoanode in a DSSC. The role of the thin film was related to the hole and electron conductivity. To begin, we can focus on the nature of the hybrid under analysis, which basically was a carboxylic acid-functionalized multi-walled carbon nanotubes-polyindole/Ti_2_O_3_ nanocomposite (f-MWCNTs-PIN/Ti_2_O_3_). The route that led to such new materials began with the functionalization of MWCNTs by means of a strong oxidizing solution composed of HNO_3_/H_2_SO_4_. The newly introduced carboxylic acid functionalities on the CNS allowed the reaction with indole. A schematic illustration is reported in Figure 8.

As it appears in the image, the smooth surface of the f-MWCNT did not allow the deposition of the indole, and consequently, the growth of polyindole (PIN) began preferentially on the surface of Ti_2_O_3_ due to the high surface area and the different chemical activity. The so-synthesized hybrid material could be rationalized into a wire of Ti_2_O_3_ nanoparticles functionalized with poly-indole and interconnected by the carbon nanotubes. The enhanced photocatalytic activity can be seen from its application in a DSSC sensitized with Di-tetrabutylammonium cis-bis(isothiocyanato)bis(2,2′-bipyridyl-4,4′-dicarboxylato)ruthenium(II) (known as N719) dye: the experimental results showed that the photovoltaic performance of the DSSCs was improved from 8.15 to 8.65% in comparison to a reference photoanode.

The strong interest in the use of CNSs in general to improve performances of DSSCs is indeed indicated by a quick increase in reports in the past year [76,77,78,79].

## 4. Perovskite and Other Hybrid and Organic Photovoltaics

Organic and hybrid photovoltaics, based on sandwiching an active layer between two electrodes capable of generating a charge once photoexcited, are experiencing ever greater development in recent years thanks to the increasing efficiency of the devices [80,81]. For a detailed description of the operating principles, see the reviews [82,83].

Considerable progress has been achieved by both developing new materials for the active layers; for example, non-fullerenic acceptor systems [84] in organic photovoltaics (OPV) or the use of perovskites in hybrid cells (PSC) [85], and also by engineering the interfaces with the electrodes using layers that can act as HTMs (hole-transporting materials) or ETMs (electron-transporting materials) [86,87,88].

Thanks to their versatility, carbon-based nanostructured materials can be used both as active materials and as charge-regulating layers in both OPV and PSC cells [89,90,91]. The major limit of CNS-based materials is their poor solubility, and consequently the poor optical and morphological quality of their films. To overcome this problem, both covalent and a non-covalent functionalization approaches toward CNSs have been proposed.

**Figure 9:**
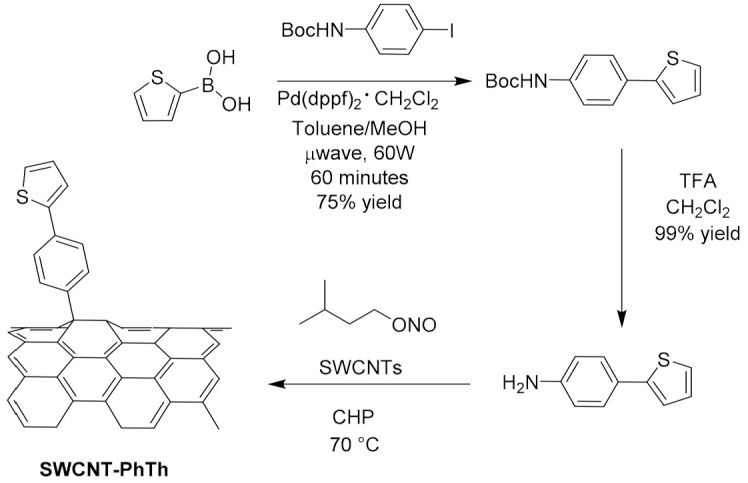
Functionalization of SWCNTs via the addition of the diazonium salt of 4-(thien-2-yl)aniline. Reproduced from [96] with the permission of the Royal Society of Chemistry.

Considering covalent functionalization, in 2017, Salice et al. [92] proposed CNT derivatization via a Tour reaction. In particular, beginning with the consideration that thienyl group increases the interaction between CNT and the P3HT used as active layer in OPV, they synthetized the 4-(thien-2-yl)-aniline that was reacted with isopentylnitrite in 1-cyclohexyl-2-pyrrolidone for the in situ generation of the diazonium salt used for the derivatization of the CNTs. SWNCTs (Figure 9) produced by the HiPco [93,94] process were functionalized by simple addition of the diazonium salt of the aniline derivative with two different approaches: one based on the traditional reactions in a flask at 80 °C for 4 h, and the other based on flow reactors by setting a flow rate of 8.0 mL/h and a temperature of 80 °C, since such reactors offer good productivity and increased safety and control of the reaction parameters with respect to traditional synthetic procedures [95].

The functionalized materials at the end of the addition reaction were precipitated by centrifugation after addition of methanol. For the preparation of solution-processable SWCNT/P3HT blends, the soluble fraction of the SWCNT-PhTh derivative was isolated by extraction with chlorobenzene and sonicated, and the supernatant was collected after the precipitation of less-soluble material.

Importantly, the study of different P3HT:CNTs heterojunctions evidenced that an excessive modification of the CNTs’ surfaces, due to the high number of carbon atoms converted from sp^2^ to sp^3^ after the functionalization, resulted in a decrease in the electronic and thermal properties of the pristine CNTs. Moreover, an uncontrolled functionalization shielded the surfaces of the CNTs, thus preventing the interaction with P3HT. The use of flow methods for CNT functionalization has allowed researchers to obtain derivatives with a good compromise between processability and retention of the SWCNT properties required for an electronic interaction with P3HT.

The covalent functionalization via a Tour reaction of SWCNTs and RGO has been proposed [97,98] for the development of HTMs in PSCs. The literature data indicates that CNSs have a double ability to increase both efficiencies and stabilities of PSC devices. In fact, CNSs “shield” the perovskite film from atmospheric moisture adsorption and thermal degradation [90,99], and at the same time, they facilitate hole extraction from the perovskite layer, [100,101] if compared to usual HTMs as Spiro-OMeTAD [102].

In this framework, SWCNTs and RGO, covalently functionalized with *p*-methoxyphenyl substituents (to obtain the derivatives SWCNT-PhOMe and RGOPhOMe, respectively) embedded in P3HT HTMs have been proposed [97]. The functionalization proceeded through the in situ generation of the diazonium salt of *p*-methoxyaniline in the presence of isopentylnitrite, using 1-cyclohexyl-2-pyrrolidone (CHP) as the solvent. The relative amounts of reactants, together with the reaction times, were regulated to avoid an excessive functionalization of the CNSs’ derivatives, preserving their electronic properties.

The electronic structure of the pristine SWCNTs was preserved, as confirmed by the absorption spectra, and an FD of 5% for SWCNT-PhOMe and 3.4% for RGOPhOMe was estimated through the TGA.

**Figure 10:**
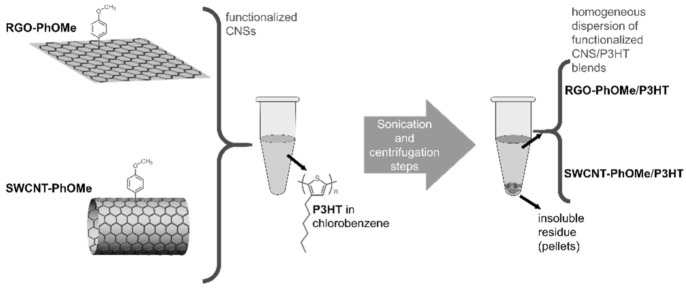
Schematic representation of the sedimentation-based separation process used for the preparation of the *p*-methoxyphenyl-functionalized CNS/P3HT blends. Reproduced from [97] with the permission of John Wiley and Sons.

Ultrasonication followed by centrifugation steps have been reported to blend SWCNT-PhOMe or RGO-PhOMe in a P3HT matrix (see Figure 10). The centrifugation steps allowed the removal of the insoluble residues, and homogeneous blends of functionalized CNSs and P3HT in chlorobenzene, an orthogonal solvent for the deposition of P3HT-based HTMs on a perovskite layer, were reported. The percentage in weight of *p*-methoxyphenyl-functionalized CNSs with respect to the P3HT was determined by weighting the pellets after the removal of the supernatant, resulting in 3 and 4 wt % for the SWCNT- and RGO-PhOMe/P3HT, respectively.

The designed HTMs were tested in PSCs, and showed an enhancement in photovoltaic performances with respect to PSCs based on simple P3HT. The CNS-P3HT-based HTMs increased the PSCs’ stability. In fact, ageing tests, carried out over 3240 h, showed an η average of 8.7% and 4.7% for SWNTs and RGO-based PSCs, respectively.

**Figure 11:**
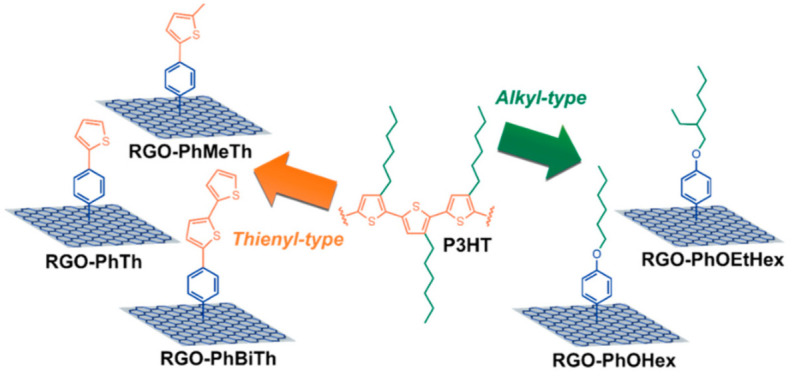
Schematic illustration of the five types of functionalized RGO species reported in this work, highlighting the similarity of relationships existing among the organic substituents covalently bound to RGO and the P3HT structure. Reproduced from [98] with the permission of Wiley.

Five types of organic moieties similar to the polymeric backbone and alkyl side chains of P3HT were covalently bound to RGO by Gatti et al. [98] to further improve the effectiveness of the functionalization for RGO@P3HT HTMs in PSCs. The proposed moieties were 4-(thien-2-yl)phenyl (PhTh), 4-(5-methylthien-2-yl)phenyl (PhMeTh), and 4-[(2-20-bithiophene)-5-yl]phenyl (PhBiTh) residues, containing thienyl groups recalling the polymer backbone; and the 4-(hexyloxy)phenyl (PhOHex) and 4-[(2-ethyl)hexyloxy]phenyl (PhOEtHex) residues, containing alkyl chains, as in the polymer side-chains (see Figure 11).

In situ direct arylation between the corresponding aniline derivative and isoamyl nitrite was used for the synthesis of the organic derivatives. The different reactivity of the aniline precursors led to different FDs, from 1.2% in RGO-PhTh to 3.2% in RGO-PhOHex. The five types of functionalized RGO materials were dispersed in P3HT, beginning with a suspension of functionalized RGO/P3HT (1:10 wt/wt; i.e., 10 wt %) in chlorobenzene. After removal the insoluble residue, the composite films with the different RGO derivatives were casted from final solutions with different wt % contents, all exceeding the 4 wt % threshold. The chemical nature of the functional groups grafted to RGO was revealed to be crucial in driving PSC efficiency. In fact, the P3HT blend containing functionalized-RGO filler with hexyl chains was found to outperform the PCE and the reproducibility of the other type of composite HTM based on bithienyl-decorated RGO, and this behavior was attributed to the different morphology of RGO flakes.

**Figure 12:**
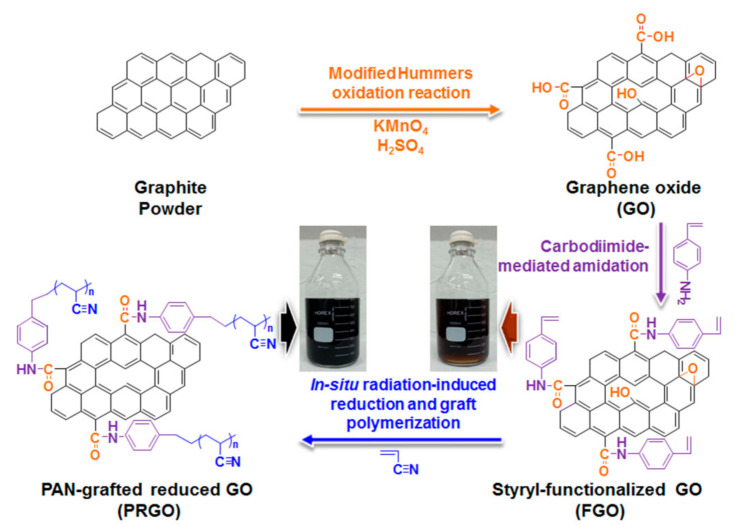
Schematic illustration for the synthesis of PRGO (insets represent the photograph of the respective FGO and PRGO solutions in 800 mL DMF). Reproduced from [103] with the permission of Elsevier.

Recently, it has been reported that an aliphatic polyacrylonitrile-grafted reduced graphene oxide (PRGO) hybrid can function as hole-extraction layer (HEL) in OPVs and PSCs [103]. The hybrid-structure PRGO was developed by in situ radiation-induced reduction and graft polymerization with polymerizable styryl-functionalized graphene oxide and acrylonitrile. The synthesis of PRGO was sequentially performed as shown in (Figure 12). GO was synthesized from the graphite powder using the well-known modified Hummers method [104,105], and was subsequently functionalized through a carbodiimide-mediated coupling reaction between the COOH groups of the GO and the amine of 4-aminostyrene at room temperature to obtain a polymerizable styryl-functionalized GO (FGO) [106]. Finally, a homogenous mixture solution of FGO and acrylonitrile (as a grafting monomer) in dimethylformamide (DMF) was subjected to γ-irradiation at room temperature to produce highly dispersible PRGO. The active electrons formed from the radiolysis of DMF could deoxygenate FGO and simultaneously initiate the graft polymerization of acrylonitrile at the introduced styryl groups [107,108,109].

**Figure 13:**
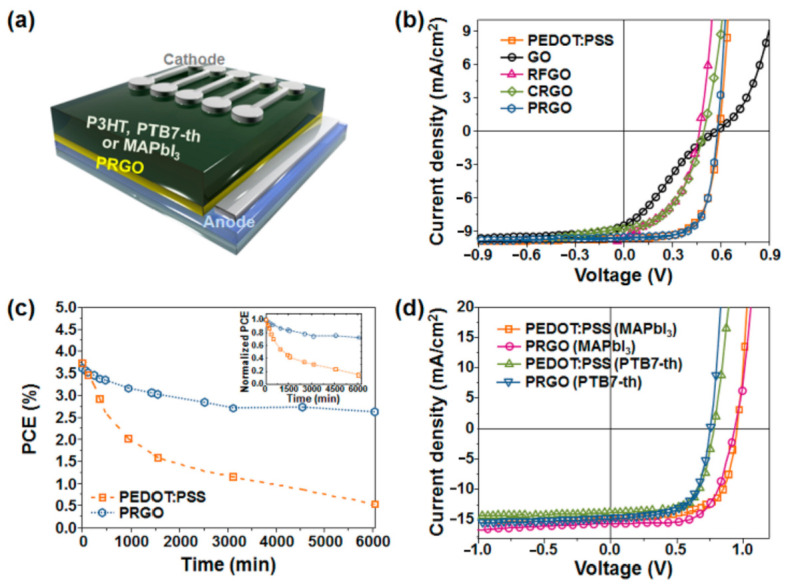
(**a**) Schematic configuration of P3HT-, PTB7-Th-, and MAPbI3-perovskite-based PVs with PRGO as a HEL; (**b**) representative J-V characteristics of P3HT-based devices with different HELs of PEDOT:PSS, GO, RFGO, and PRGO; (**c**) PCE decay of P3HT-based PVs with different HELs of PEDOT:PSS and PRGO as a function of exposure time to the ambient atmosphere (the inset shows the normalized PCEs); (**d**) representative J-V characteristics of PTB7-Th- and CH3NH3PbI3-perovskite-based PVs with different HELs of PEDOT:PSS and PRGO. Reproduced from [103] with the permission of Elsevier.

The final PRGO solution had a typical black color and a grafting degree ranging from 22 to 42 wt % was obtained by measuring the weight of PRGOs before and after the cleavage reaction of polyacrylonitrile chains from the PRGOs, indicating that the grafting degree of PAN could be controlled by the grafting conditions of the monomer concentration and absorbed dose. The formation of the covalently hybrid-structured PRGO was confirmed by Fourier transform infrared spectroscopy (FT-IR), X-ray photoelectron spectroscopy (XPS), X-ray diffraction spectroscopy (XRD), transmission electron microscopy (TEM), dynamic light scattering (DLS), Raman, ultraviolet–visible spectroscopy (UV–vis), and thermogravimetric analysis (TGA). The comparative analysis confirmed that the polymerizable styryl group was bound to the COOH of GO, and that PRGO was successfully synthesized via γ-ray irradiation-induced reduction of FGO and simultaneous graft polymerization of acrylonitrile at the styryl groups. The PRGO hybrid material displayed good dispersion stability of six months, even at a high concentration of 10 mg/mL, with good film morphology, an electrical conductivity of 0.87 S/cm, a work function of 4.87 eV, and weather stability. A PCE of 7.24% for a PTB7-Th-based OPV and 9.70% for a MAPbI3-based PSC was measured (see Figure 13) with the incorporation of PRGO as a HEL; these values were comparable to those from the PEDOT:PSS-based PVs, but with increased device stability.

Parallel to the covalent functionalization approach, new hybrid materials exploiting the non-covalent functionalization strategy have been developed.

**Figure 14:**
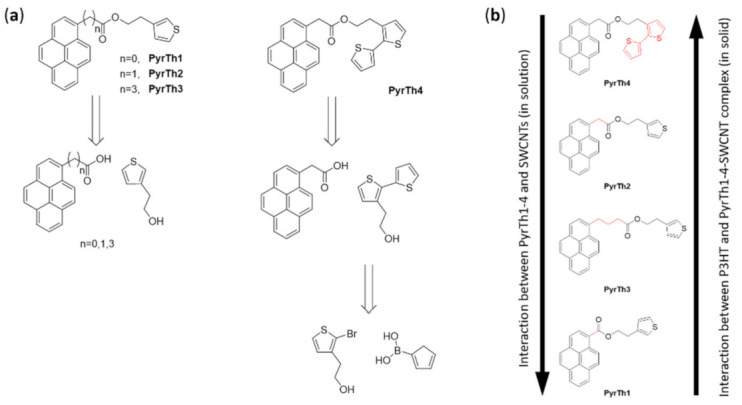
(**a**) Retrosynthetic approach to the preparation of thiophene derivatives with pyrene pendants: esterification of 3-thiopheneethanol (for derivatives PyrTh1–3) and 3-bitiopheneethanol (for derivative PyrTh4 with carboxylic derivatives of pyrene). Reproduced from [92] with the permission of Elsevier. (**b**) Qualitative comparison of the pyrenyl derivatives’ interaction with SWCNTs as observed by fluorescence quenching of pyrenyl derivatives in SWCNT solution and P3HT/pyrenyl-derivatives/SWCNTs thin solid films.

For this reason, the development of suitable organic molecules able to improve the interaction with organic semiconductors represents a very important objective. In 2017, Sartorio and coworkers [92] proposed a series of pyrenyl derivatives as suitable materials to tailor the interaction between SWCNTs and P3HT to be used for electron transfer in thin-film heterojunction, with P3HT as the donor system. They developed four bifunctional organic derivatives (PyrTh1-4) composed of a pyrene moiety able to interact with the CNTs’ carbon shell and a thiophene unit to improve the affinity with P3HT. The 3-thiopheneethanol, 3-bithiopheneethanol, and commercially available pyrene derivatives were selected to obtain the ester derivatives via Steglich esterification with 1-ethyl-3-(3-dimethylaminopropyl)carbodiimide and 4-dimethylaminopyridine (reported in Figure 14).

The supramolecular interaction in solution was verified via solution fluorescence measurements able to provide a means to assess the nature (static or dynamic) and the strength of the interaction between CNTs and the bifunctional pyrene–thiophene derivatives in solution.

The analysis of the quenching constants suggested that the interaction with SWCNTs was mainly due to the π–π interactions of pyrene units with the nanotubes’ surfaces. The length of the alkyl chains was not relevant, while the ester moiety provided a repulsive contribution. The effectiveness of the supramolecular approach was confirmed in a solid state by XPS and AFM. The first was used to analyze the chemical composition, confirming a good amount of pyrene derivatives per carbon nanostructure, while the AFM confirmed the presence of isolated nanotubes.

**Figure 15:**
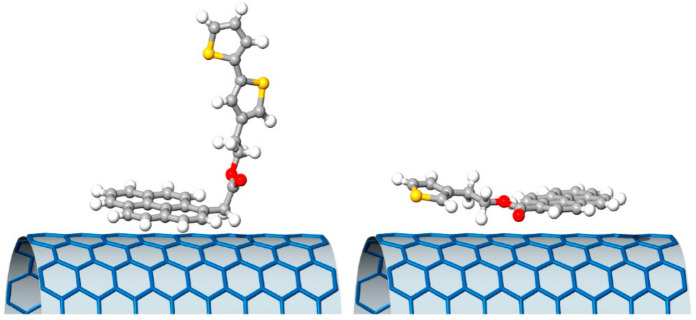
Schematic drawing of the ground state minimum of PyrTh4 (**left**) and PyrTh1 (**right**), and of their possible different approaches to the surface of a carbon nanotube. Reproduced from [92] with the permission of Elsevier.

The weakest interaction with the SWCNTs was obtained for the compound PyrTh1, in which ester and pyrene were coplanar, while the stronger interaction was obtained for PyrTh4 and PyrTh2, in which the π–π interactions between pyrene and carbon nanostructures were not affected by the presence of the ester group (Figure 14 and Figure 15). As a result, good processability and efficient electron transfer were obtained in thin-film heterojunctions system in which the alkyl chain was coplanar with the pyrene moiety.

**Figure 16:**
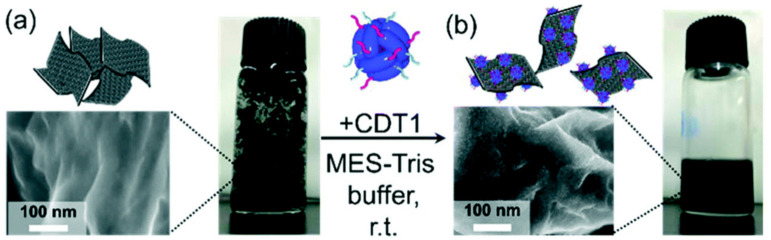
FE-SEM images and photographs of (**a**) graphene and (**b**) CDT1 with the iron oxide core adsorbed on the graphene. These materials were dispersed in water. Reproduced from [110] with the permission of the Royal Society of Chemistry.

A supramolecular green approach was proposed by Hashima et al. [110] for the preparation of a graphene/TiO_2_ nanohybrid. They proposed an artificially bifunctionalized protein to increase the dispersibility of the graphene in water. A variant protein (carbonaceous material binding peptide-Dps-titanium binding peptide (CDT1)) [111,112] derived from a Listeria innocua Dps protein synthesized from encoded Escherichia coli was employed [104,113,114,115,116]. Dps (a DNA-binding protein from starved cells) [117,118] is a cage-shaped protein able to generate inorganic oxides in the inner and outer cavity by biomineralization. The introduction of peptides on the outer surface increased the interaction with graphene, and aromatic amino acid parts could bind graphene through π–π interactions, while carboxylates could coordinate with Ti derivatives on CDT1, promoting the generation of the graphene/TiO_2_ hybrid. The graphene/CDT1 complex was prepared by simply mixing graphene and CDT1 in a buffer solution via sonication with subsequent washing and centrifugation of the precipitate. The FE-SEM image of the precipitate using a CDT1 with an iron oxide showed the nanosheet structure and 4 nm dots on the surface of the graphene, which was attributed to CDT1 with iron oxide adsorbed on the graphene surface. (Figure 16) The amount of adsorbed CDT1 on the graphene was quantified by Klotz plots together with the dissociation constants [119], and the adsorption phenomena were attributed to the electrical surface conditions of CDT1 at an optimal pH of around 6.0. The nanohybrid of graphene/CDT1 and TiO_2_ was prepared by depositing the TiO_2_ layer on the surface of the graphene/CDT1 complex using TiO_2_ as a precursor (titanium(IV)bis(ammonium lactato)dihydroxide) Ti[BALDH] that was biomineralized by the protein.

**Figure 17:**
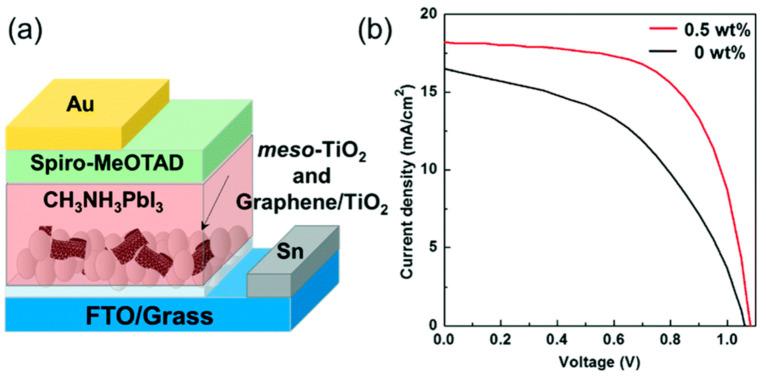
(**a**) An illustrated structure of the fabricated PSC. (**b**) I–V characteristics of PSCs with (red line) or without (black line) the graphene/TiO_2_ nanohybrid in the meso-TiO_2_ layer. Reproduced from [110] with the permission of the Royal Society of Chemistry.

Transmission electron microscopy (TEM) and elemental mapping via electron energy-loss spectroscopy confirmed the nanohybrid formation. PSCs were fabricated using the composite at the interface between FTO electrode and perovskite. The nanohybrid (0.5 wt %) was mixed with a meso-TiO_2_ paste by a sintering process at 450 °C in which the CDT1 was completely burned and the final performance of the device was higher than that of the PSC without the nanohybrid, most probably due to the prevention of electron–hole recombination (see Figure 17).

The continuous development of organic electronics and the need to replace metals, which are non-renewable raw materials, with more abundant and renewable materials has led to the demand for transparent conductive electrode (TCE) alternatives to indium tin oxide (ITO), which is composed of non-renewable raw materials [120]. Graphene is a very promising alternative material for TCEs, thanks to its excellent electrical and optical properties, and as a carbon-based material that is renewable and with a potentially low cost.

The major drawback related to graphene’s development is due to its inert nature, which is responsible for its poor wetting of charge-transporting materials. Jung S. et al. [121] proposed to use norepinephrine [122], a derivative of catecholamine composed of a hydrophobic benzene ring and hydrophilic functional groups, as a coating for a graphene electrode to increase the wettability.

**Figure 18:**
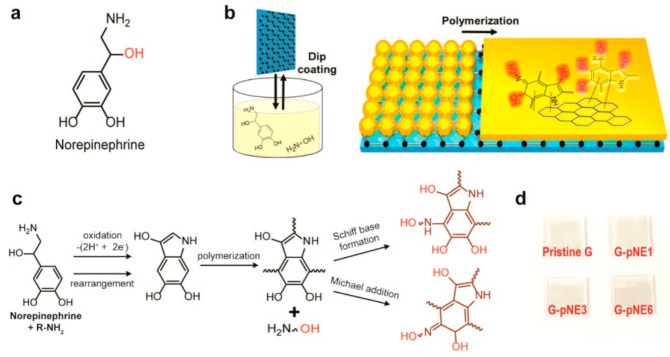
Formation of the polyNE film on graphene. (**a**) Molecular structure of norepinephrine. (**b**) Schematic of the process of coating and polymerization of norepinephrine on graphene. (**c**) Scheme for the polymerization pathway of norepinephrine. (**d**) Digital image of pristine and pNE-coated graphene on glass substrates. Reproduced from [121] with the permission of the American Chemical Society.

The graphene film surface was modified via a catechol oxidative polymerization [123,124] by film immersion in a pH 8.5 buffer solution containing norepinephrine (NE) and 2-(2-aminoethoxy)ethanol, which is a hydrophilic primary amine. The intermolecular cross-linking of catechol derivatives was suppressed thanks to the use of a primary amine reacting with oxidized catechol, forming catecholamine adducts via Schiff base formation or Michael addition reaction, resulting in the formation of a smooth thin film of polyNE. Moreover the hydroxyl group in the 2-(2-aminoethoxy)ethanol molecule added an extra hydroxyl group to the polyNE coating layer, increasing the hydrophilicity of the surface as confirmed by the water contact angle (Figure 18).

**Figure 19:**
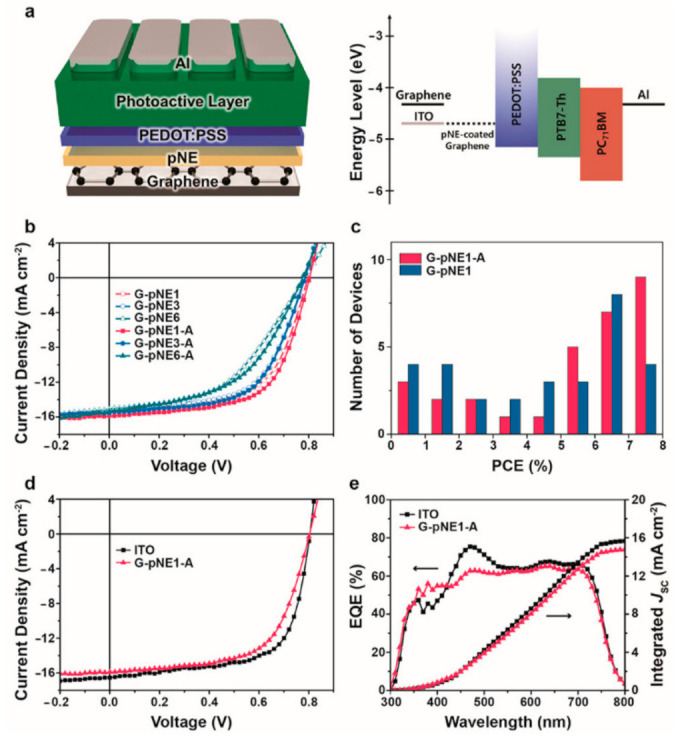
Device performance of the pNE-coated graphene-based OSCs. (**a**) Schematic of the conventional device and the corresponding flat-band energy-level diagram. (**b**) J−V characteristics of the pNE-coated graphene-based OSCs with and without the annealing treatment. (**c**) Performance statistics for the OSCs with and without annealing. (**d**) J−V characteristics and (**e**) EQE measurements with corresponding integrated Jsc of the device with the best performance (G-pNE1-A) compared with those of the ITO reference. Reproduced from [121] with the permission of the American Chemical Society.

For device applications, a film thickness of less than 10 nm was used to minimize the insulating effect of the polyNE, and an annealing treatment to improve the film quality was performed. The thermal treatment did not cause a significant change in chemical composition, and improved the overall conductivity of the film by reducing the vacant spaces and improving the contact between the graphene and polyNE. The modified hydrophilic graphene surface allowed good coverage of a conventional HTM as PEDOT:PSS, and as a result, efficient graphene-based OSCs were prepared with a performance comparable to that of the ITO reference device, as reported in Figure 19.

## 5. Photocatalytic Fuel Production

Using solar energy to produce fuels (typically hydrogen) can be a convenient route to reduce pollution and carbon footprint, bringing the advantage of storing energy in a stable way until it will be consumed; for example, in electric vehicles equipped with fuel cells [125]. CNSs have been proposed for years as components of photocatalytic systems for the production of H_2_, based on both inorganic semiconductors and organic dyes [8,126].

The most recent investigations in the field were focused on hybrid photocatalysts utilizing graphene-based materials. Porphyrins and phthalocyanines, due to their intense absorption in the visible-light region and efficient photoinduced electron-donation ability, are very promising organic photoactive moieties for hydrogen evolution. Moreover, their structure offers the possibility to form stable bonds with CNSs, either covalent or non-covalent.

Among graphene-based materials, GO offers a plethora of oxygen-based functional groups for the grafting of molecules. Oxygen itself can coordinate the metal of a porphyrin complex, such as in the example reported by Ping and coworkers [127]. The authors synthesized a hybrid material with a manganese tetraphenylporphyrin (MnTPP) covalently linked to GO by simply reacting MnTPPCl and GO at 85 °C for 5 days in pyridine. The covalent bond was confirmed by XPS, FTIR (showing a peak for Mn–O vibration) and Raman (with a red-shift of D and G bands due to the attached electron donor porphyrin). Under UV–vis irradiation in water, the system exhibited significant H_2_ evolution (3.8 μmol mg^−1^ after 6 h of UV–vis irradiation). The system was then modified by depositing Pt nanoparticles on GO, through in situ photoreduction of a H_2_PtCl_6_ solution, leading to an enhancement of photocatalytic activity (4.6 μmol mg^−1^), thanks to a lowering of the electrochemical overpotential. A further enhancement of hydrogen evolution (5.2 μmol mg^−1^) was achieved after addition of polyvinyl pyrrolidone (PVP) as surfactant, leading to a more homogeneous dispersion and to a larger available active surface.

**Figure 20:**
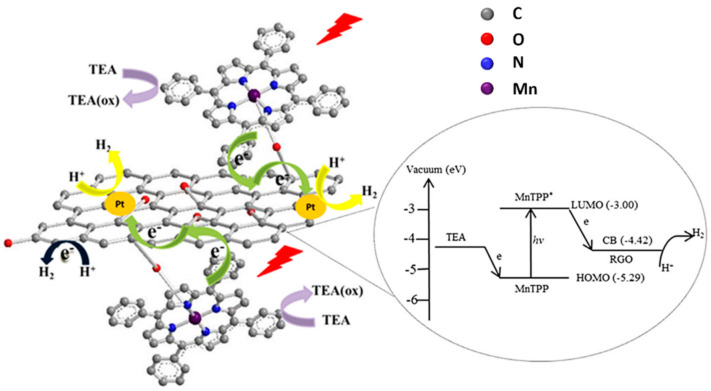
Diagram of the proposed photocatalytic mechanism for GO-MnTPP/Pt. Reproduced from [127] with the permission of World Scientific.

Time-resolved optical spectroscopy suggested a rapid photoinduced electron transfer from the sensitizer molecule MnTPP to GO, allowed by the direct linkage (see Figure 20). The proposed mechanism involves a subsequent electron transfer to Pt nanoparticles, where H^+^ is reduced to H_2_. The oxidized porphyrin moiety is then regenerated by a sacrificial donor (TEA = triethylamine).

Using GO as supporting matrix, given its excellent properties as an electron mediator, enhances electron-transfer processes and prevents charge recombination.

Carboxylic groups, ubiquitous in the GO structure, can be used to graft amine-functionalized porphyrins through amide bond formation [128]. Following this approach, Yan and coworkers functionalized GO with an aminated tetraphenylporphyrin (TPP-NH_2_) photosensitizer [129]. To activate GO, it was refluxed in SOCl_2_ in the presence of DMF for 24 h under argon. After purification, the product was reacted with TPP-NH_2_ in DMF at 130 °C for 72 h under argon. In addition, [FeFe]-hydrogenase model (IHM) complexes (as proton-reduction catalysts) were linked to the basal plane of GO through non-covalent π–π interactions with the aromatic ring of the complex, inspired by a previous work on biomolecular photocatalysts [130].

**Figure 21:**
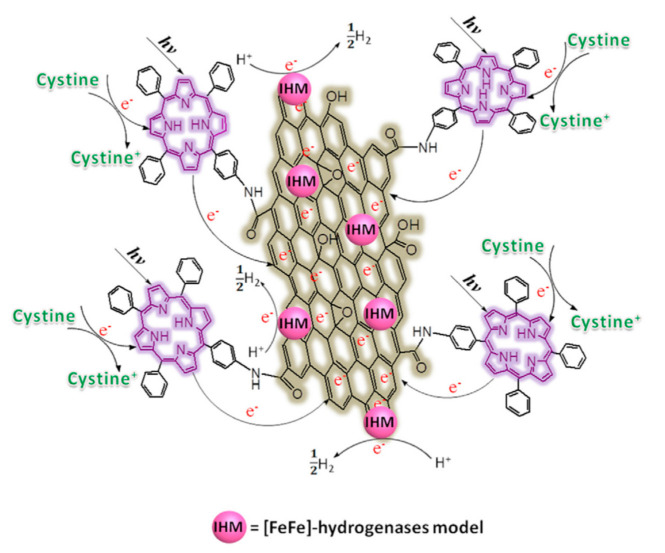
The proposed mechanism of photocatalytic H_2_ production for a catalytic system based on GO, a porphyrin sensitizer and a biomimetic catalyst. Reproduced from [129] with the permission of Elsevier.

The biomimetic photocatalytic system resulting from the double decoration benefits from the electron mediation operated by GO between the photosensitizer and the catalytic unit, thus showing enhanced hydrogen evolution. Moreover, GO hydrophilicity improves dispersion of TPP and of the catalytic complex in water/ethanol. A schematic representation of the proposed photocatalytic H_2_ production mechanism is reported in Figure 21. Cystine was chosen as a sacrificial donor to simulate a biological protein environment. The proposed working principle also was supported by emission spectroscopy, showing fluorescence quenching by 80.6% when TPP was bound to GO, probably due to electron transfer from the dye to the nanostructure. The pivotal role of covalent functionalization with the organic sensitizer was highlighted by control experiments in which the porphyrin was just adsorbed on GO, affording less-efficient hydrogen production.

**Figure 22:**
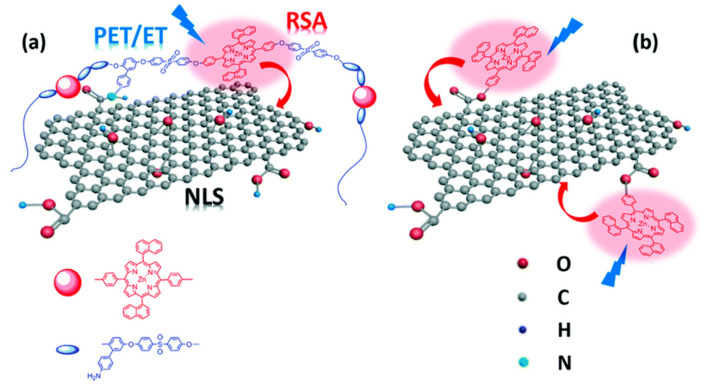
Schematic representation of structures and photoactivity for PF–GO (**a**) and ZnP–GO (**b**). Reproduced from [131] with the permission of the Royal Society of Chemistry.

The use of amide bonds also was reported by Wang and coworkers, in a study focused on materials with non-linear optical properties that also could be promising candidates for photocatalytic hydrogen generation, although this was not proved [131]. The authors grafted a porphyrin-based polymer (ZnTNP–PAES) to the carboxylic groups on GO to obtain a photoactive hybrid (PF–GO; see Figure 22). They also used a similar strategy, but based on ester bonds, to functionalize GO with 5-(4-hydroxyphenyl)-10,15,20-trinaphthylporphyrin zinc (ZnTNP–OH) to obtain ZnP–GO (see Figure 22b).

ZnTNP–PAES is a copolymer of phenyl sulfone, (*p*-amino)-phenylhydroquinone, and a symmetrical zinc dinaphthylporphyrin. Aminated moieties on the copolymer are reacted with COOH groups on GO to form amide bonds, while the abundant aromatic rings in the backbone can establish strong π–π interactions with GO basal plane, thus stabilizing the hybrid, preventing aggregation of the nanosheets and improving their dispersion. To obtain the hybrids, the authors first activated GO through reaction with SOCl_2_. The obtained acyl chloride derivative GO–COCl was then reacted in anhydrous DMF and trimethylamine at 80 °C for 72 h under argon, with ZnTNP–PAES or with ZnTNP–OH to obtain PF–GO or ZnP–GO, respectively. Chemical stability of porphyrins and related structures allowed a further chemical transformation of the hybrid. Indeed, PF–GO was reduced with hydrazine to obtain the corresponding RGO/polymer hybrid PF–RGO.

Photophysical investigations of porphyrin hybrids, compared to starting polymer and porphyrin, revealed features such as relevant fluorescence quenching (65% and 57% for PF–GO and ZnP–GO, respectively, and even more for RGO hybrids) and decreased lifetimes of excited states that are indicative of energy- or electron-transfer processes, thus opening the possibility to consider them for solar energy conversion, and in particular for photocatalytic applications in analogy with other porphyrin-CNS derivatives [132].

**Figure 23:**
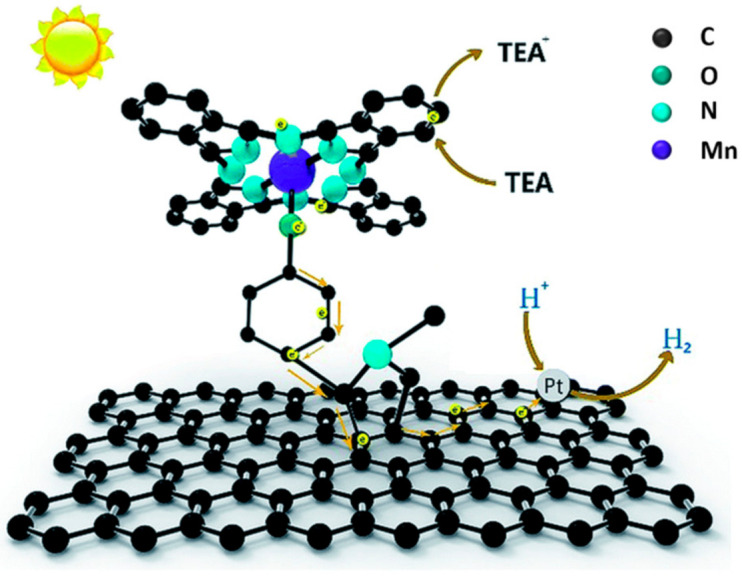
Schematic representation of MnPcG complex and of its photocatalytic activity in the presence of Pt nanoparticles and triethylamine (TEA) as sacrificial reducing agent. Reproduced from [133] with the permission of the Royal Society of Chemistry.

Another covalent approach, based on the Prato reaction, was used by Lu and coworkers to prepare a hybrid material with a manganese phthalocyanine grafted to graphene, which in turn was obtained through mechanical exfoliation of graphite [133]. In detail, the 1,3 dipolar cycloaddition reaction with *N*-methyl-glycine and 4-hydroxybenzaldehyde afforded a graphene–pyrrolidine derivative with a phenol group that, after OH deprotonation with K_2_CO_3_, was reacted with the manganese phthalocyanine chloride to form the hybrid complex MnPcG (represented in Figure 23) through formation of a Mn–O bond.

An efficient photocatalytic hydrogen production from water was observed (7.52 μmol mg^−1^ after 10 h of UV irradiation), and the importance of covalent functionalization was highlighted by comparison with a simple mixture of graphene and manganese phthalocyanine chloride, which afforded 7.52 μmol mg^−1^. As in the case of GO-MnTPP, hydrogen evolution further increased after decoration of graphene sheets of the complex with Pt nanoparticles, through in situ photoreduction of H_2_PtCl_6_. A similar photocatalytic mechanism to that of GO-MnTPP/Pt was proposed, as depicted in Figure 23, and also in this case, the CNS played the role of a substrate supporting the organic sensitizer and enhancing electron transfer to the catalytic site, thanks to superior electronic properties offered by the sp^2^ carbon lattice. Moreover, the overall structure of the covalent hybrid seemed to contrast recombination, thus extending the lifetime of photogenerated separated charges, and eventually improving hydrogen reduction efficiency.

Notwithstanding the relevance of covalent linkages between sensitizers and CNSs, large aromatic systems such as porphyrins also offer the possibility of relatively strong non-covalent interactions with the basal plane of graphene-based materials. This was the case for the hybrids obtained by Lewandowska et al. by mixing a GO solution in water and a porphyrin solution in THF [134]. The resulting π–π stacking interactions accounted for the observed quenching of fluorescence, but they left other properties of the porphyrin unaffected. Overall spectroscopic evidence, including EPR measurements and density functional theory (DFT) calculations, suggested that the porphyrin–GO complex can be used as an active material for applications based on photoinduced electron-transfer processes.

**Figure 24:**
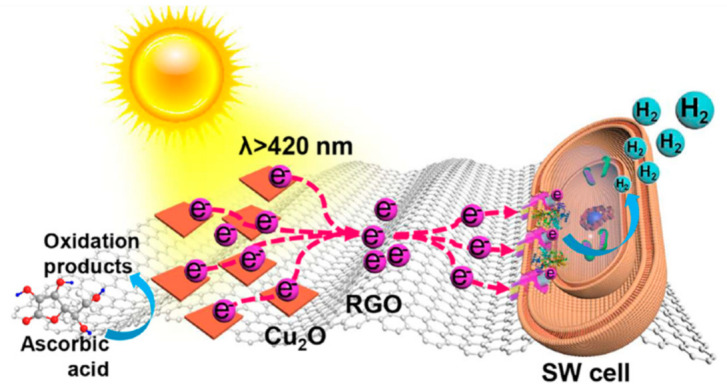
Schematic illustration of charge separation and transfer giving rise to hydrogen evolution under visible light irradiation in an RGO-based bio-hybrid including Shewanella oneidensis (SW) bacteria. Reproduced from [135] with the permission of the American Chemical Society.

A similar non-covalent interaction involving RGO and a heme moiety was proposed as the basis of a bio-hybrid system, obtained by Yong and coworkers, showing photocatalytic H_2_ production [135]. In this case, the organic molecular component was replaced by whole bacterial cells (Shewanella oneidensis) deposited on RGO sheets previously decorated with Cu_2_O clusters (Figure 24). Supported by time-resolved photoluminescence investigations and DFT calculations, the authors detected electron-transfer processes, enabling photocatalytic hydrogen evolution enabled by hydrophobic interactions between RGO and membrane-bound redox proteins (MtrC/OmcA), and in particular their heme moiety.

The proposed mechanism involved a photoinduced charge separation at Cu_2_O and a subsequent electron flow, mediated and enhanced by RGO, to the redox proteins embedded in cell membranes, where hydrogen reduction takes place. Indeed, photocatalytic H_2_ production efficiency of the bio-hybrid (322.0 μmol/g_Cu2O_ after 4 h of visible-light irradiation) was at least 11 times higher than model Cu_2_O/bacteria hybrids without RGO. Moreover, control experiments with deletion of the MtrC/OmcA proteins in the cells afforded no H_2_ evolution, thus confirming the proposed photocatalytic mechanism.

Even inside the cavity of an SWCNT, a photoactive molecule such as a ferrocenyl dye can absorb light and give rise to photoinduced electron transfer to a fullerene derivative (a water-soluble fullerodendrimer [136]) adsorbed on the outer side of the wall, as reported by Takaguchi and coworkers [137]. Encapsulation was achieved by refluxing SWCNTs and the ferrocenyl derivative in 1,2-dimethoxyethane for 3 h, followed by filtration and careful washing of the product to remove any molecule sticking to the outer wall of the tubes. Non-covalent adhesion of the fullerene derivative was then achieved by sonicating a dispersion of the endohedral SWCNT derivative in a water solution of the fullerodendron for 4 h at room temperature. The obtained hybrid acted as a photosensitizer in the presence of methyl viologen (MV) cations, 1-benzyl-1,4-dihydronicotinamide (BNAH), and Pt nanoparticles. Upon photoexcitation, the encapsulated dye donated one electron to the fullerene, while the hole left over on the dye was transferred to the SWCNT. Mediated by the couple MV^2+^/MV^+^, the electron was then transferred to the Pt nanoparticle, where it reduced H^+^ to H_2_. On the other side, BNAH acted as a sacrificial reducing agent, and donated one electron to the SWCNT to regenerate the photocatalytic system. Hydrogen generation in this case was only used as a proof of concept to demonstrate, for the first time, a PET occurring through the wall of a SWCNT. Once again, functionalization played a relevant role in a system based on different non-covalent interactions between a SWCNT and two molecules, inside and outside the tube. The same team very recently reported an evolution of this system, in which the nanopeapods were rendered dispersible in water through non-covalent exohedral functionalization with solubilizing dendrimers [138].

**Figure 25:**
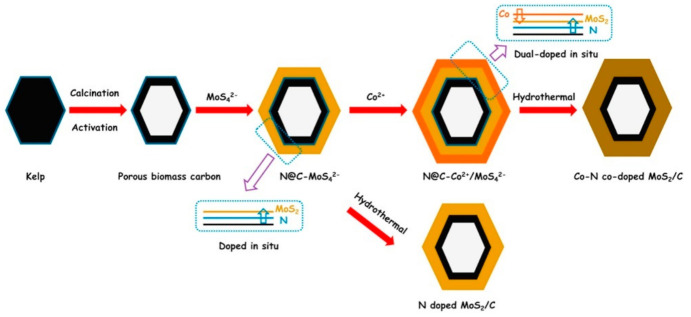
Schematic illustrations of the preparation processes of the Co/N dual-doped-MoS2/C heterostructure composite. Reproduced from [139] with the permission of Elsevier.

In recent years, researchers have been increasing their focus on the production of carbon nanomaterials derived from biomass or food waste as a sustainable approach that lowers carbon footprints and embraces circular economy paradigms. In this frame, it is worth mentioning an unusual example of a hybrid photocatalytic system for hydrogen evolution based on porous biomass carbon (PBC) [139]. In the work reported by Chen and coworkers, organic functionalization in a strict sense was not involved, but rather the organic components in the biomass (kemp) were converted into N-doped porous carbon. Natural sea kemp collected in Dalian (China), the nitrogen content of which was evaluated to be 5.84% with respect to carbon, was calcinated under a N_2_ flow at 800 °C and activated with a KOH solution, and then neutralized, rinsed, and dried. The resulting PCB substrate was first coated with a MoS^4−^ layer and then with Co^2+^ by means of solution treatments with (NH_4_)_2_MoS_4_ and Co(NO_3_)_2_·6H_2_O salts, respectively. The resulting material was then subjected to a hydrothermal treatment that afforded a PBC-supported MoS_2_ catalyst co-doped with Co and N, the latter migrated from the biomass-derived carbon (see the process scheme in Figure 25).

The peculiarity of this sustainable approach lay in the conversion of the bio-organic material into a conductive substrate that facilitated electron-transfer processes, thus leading to photocatalytic hydrogen evolution, and at the same time enabled doping of the inorganic semiconductor, since the biomass was a source of both carbon and nitrogen.

## 6. Conclusions and/or Perspectives

In this review, we have attempted to provide a comprehensive overview of the use of functionalized organic carbon nanostructures for solar energy conversion. Our attention was focused in particular on the most recent contributions by organic chemistry to the development of new hybrid materials that find applications in DSSCs, OPVs, and PSCs, and in photocatalytic fuel production.

The interest in carbon-based nanomaterials has developed thanks to their exceptional electrical/thermal conductivity, high chemical stability, and mechanical strength. Furthermore, the possibility of engineering their structures through covalent and non-covalent chemical functionalization has made it possible to correct some of their drawbacks, such as poor solubility, tendency to aggregate, and poor quality of films. Chemical functionalization is also able to modulate their optical and electronic properties by combining them with those of the materials with which they are in contact, and this makes them an even more versatile platform for new functions or components with possible synergistic effects.

The request for new materials able to accompany the green energy transition that are abundant, low-cost, and low-toxicity derived from renewable sources has further increased the interest in CNSs that meet all these requirements. In particular, as highlighted in this review, it is possible to disperse them in water, eliminating the use of toxic solvents while maintaining excellent electrical characteristics. Furthermore, their use generally leads to devices with characteristics that are equivalent, if not superior, to traditional ones, but in general, they improve the stability over time. This feature, combined with a low cost and good availability, is crucial to allow the industrial diffusion of organic and hybrid systems for energy conversion.

Specifically regarding DSSCs and focusing on an eco-friendly approach, the use of natural dyes obtainable directly from natural sources perfectly suits the aim of low-cost and environmental low-impact features of carbon nanostructure technology moving towards totally renewable sensitizers, as described in Section 3, derived from seaweeds and algae.

Green organic and hybrid electronics based on OPVs and PSCs currently represent some of the most interesting topics for the scientific community. Numerous efforts have been made to decrease the environmental impact of manufacturing these classes of devices. Currently, encouraging results have been obtained by improving production processes, eliminating chlorinated solvents, and using solution processes that allow energy savings. The use of organic functionalized CNSs perfectly meets these needs, allowing the elimination of toxic solvents; improving the performance of the devices thanks to their use as a charge-regulating layer obtained with solution techniques; and finally, increasing their stability over time, as reported in Section 4.

Furthermore, the production of carbon nanomaterials using biomass or food waste as a sustainable approach that lowers carbon footprints and embraces circular economy paradigms has been successfully exploited in photocatalytic fuel production, as described in Section 5, opening the way for many other applications, together with the implementation of bio-hybrid systems based on whole bacterial cells. In fact, the exploitation of microorganisms such as fermentative bacteria holds great promise for hydrogen generation.

So far, the selected literature works have clearly demonstrated that the inclusion of an organic molecule, thanks to both covalent and non-covalent interactions, in a carbon nanostructure leads to the formation of a completely new material that can be characterize by better spectroscopic absorbance, improved conductivity, and/or enhanced photocurrent density. In addition to the numerical data, which unequivocally state the positive effect of the new material, we hope that these examples can inspire further research in the field of photoactive materials from an organic point of view.

## Abbreviations and Acronyms

AFM	atomic force microscopy
BNAH	1-benzyl-1,4-dihydronicotinamide
CDT1	carbonaceous material binding peptide-Dps-titanium binding peptide
CHP	1-cyclohexyl-2-pyrrolidone
CNS	carbon nanostructure
CNT	carbon nanotube
DFT	density functional theory
DLS	dynamic light scattering
DMF	dimethylformamide
Dps	DNA-binding protein from starved cells
DSSC	dye-sensitized solar cell
D–π–A	donor–π–acceptor system
EPR	electron paramagnetic resonance spectroscopy
EQE	external quantum efficiency
ETM	electron-transporting materials
FD	functionalization degree
FGO	polymerizable styryl-functionalized GO
f-MWCNTs-PIN	carboxylic acid-functionalized multi-walled carbon nanotubes-polyindole
FT-IR	Fourier-transform infrared spectroscopy
FTO	glass
GBM	graphene-based materials
GDQ	graphene quantum dot
GO	graphene oxide
GQD	graphene quantum dots
H_2_SO_4_	sulfuric acid
HEL	hole-extraction layer
HiPco	high-pressure carbon monoxide
HNO_3_	nitric acid
HOMO	highest occupied molecular orbital
HTM	hole-transporting materials
IHM	[FeFe]-hydrogenase model complex
ITO	indium tin oxide electrode
Jsc	short current density
LUMO	lowest unoccupied molecular orbital
MnPcG	manganese phthalocyanine GO hybrid
MnTPP	tetraphenylporphyrin
MOCPc	metallo-octacarboxyphthalocyanines
MOF	metal–organic framework
MPC	metallophthalocyanines
MtrC/OmcA	membrane-bound redox proteins
MV	methyl viologen
MWCNT	multi-walled carbon nanotube
N719	Di-tetrabutylammonium cis-bis(isothiocyanato)bis(2,2′-bipyridyl-4,4′-dicarboxylato)ruthenium(II)
NE	norepinephrine
NH_2_-TPA-Th-H	triphenylamine-thiophene-cyan acrylic acid
NMR	nuclear magnetic resonance
OPVs	organic photovoltaics
P3HT	poly-3-hexyl-thiophene
PBC	porous biomass carbon
PCE	power conversion efficiency
PEDOT:PSS	poly(3,4-ethylenedioxythiophene)polystyrene sulfonate
PhBiTh	4-[(2-20-bithiophene)-5-yl]phenyl
PhMeTh	4-(5-methylthien-2-yl)phenyl
PhOEtHex	4-[(2-ethyl)hexyloxy]phenyl
PhOHex	4-(hexyloxy)phenyl
PhOMe	anisole
PhTh	4-(thien-2-yl)phenyl
PIN	polyindole
polyNE	poly-norepinephrine
PRGO	polyacrylonitrile-grafted reduced graphene oxide
PSC	perovskite solar cell
RGO	reduced graphene oxide
rGO	reduced graphene oxide
SWCNT	single-walled carbon nanotube
TCE	transparent conducting electrode
TEA	triethylamine
TEM	transmission electron microscopy
TGA	thermogravimetric analysis
Ti[BALDH]	titanium(IV)bis(ammonium lactato)dihydroxide)
TiO_2_	titanium dioxide
TPA-Et	methyl-2-cyano-3-(4-(diphenylamino)phenyl)acrylate
TPP	tetraphenylporphyrin
TPP-NH_2_	tetraphenylporphyrin
UV–vis	ultraviolet–visible spectroscopy
UV–vis–NIR	ultraviolet–visible–near infrared
V_OC_	open circuit voltage
XPS	X-ray photoelectron spectroscopy
XRD	X-ray diffraction spectroscopy
ZnTNP–OH	5-(4-hydroxyphenyl)-10,15,20-trinaphthylporphyrin zinc
ZnTNP–PAES	copolymer of phenyl sulfone, (*p*-amino)-phenylhydroquinone, and zinc dinaphthylporphyrin
